# The Body and the Beautiful: Health, Attractiveness and Body Composition in Men’s and Women’s Bodies

**DOI:** 10.1371/journal.pone.0156722

**Published:** 2016-06-03

**Authors:** Mary-Ellen Brierley, Kevin R. Brooks, Jonathan Mond, Richard J. Stevenson, Ian D. Stephen

**Affiliations:** 1 Department of Psychology, Macquarie University, Sydney, Australia; 2 ARC Centre of Excellence in Cognition and its Disorders, Sydney, Australia; 3 Perception in Action Research Centre (PARC), Faculty of Human Sciences, Macquarie University, Sydney, Australia; University of Udine, ITALY

## Abstract

The dominant evolutionary theory of physical attraction posits that attractiveness reflects physiological health, and attraction is a mechanism for identifying a healthy mate. Previous studies have found that perceptions of the healthiest body mass index (weight scaled for height; BMI) for women are close to healthy BMI guidelines, while the most attractive BMI is significantly lower, possibly pointing to an influence of sociocultural factors in determining attractive BMI. However, less is known about ideal body size for men. Further, research has not addressed the role of body fat and muscle, which have distinct relationships with health and are conflated in BMI, in determining perceived health and attractiveness. Here, we hypothesised that, if attractiveness reflects physiological health, the most attractive and healthy appearing body composition should be in line with physiologically healthy body composition. Thirty female and 33 male observers were instructed to manipulate 15 female and 15 male body images in terms of their fat and muscle to optimise perceived health and, separately, attractiveness. Observers were unaware that they were manipulating the muscle and fat content of bodies. The most attractive apparent fat mass for female bodies was significantly lower than the healthiest appearing fat mass (and was lower than the physiologically healthy range), with no significant difference for muscle mass. The optimal fat and muscle mass for men’s bodies was in line with the healthy range. Male observers preferred a significantly lower overall male body mass than did female observers. While the body fat and muscle associated with healthy and attractive appearance is broadly in line with physiologically healthy values, deviations from this pattern suggest that future research should examine a possible role for internalization of body ideals in influencing perceptions of attractive body composition, particularly in women.

## Introduction

Sexual Strategies Theory proposes that face and body attractiveness judgements reflect psychological adaptations for identifying healthy and fertile potential mates [1,2], with the most attractive individuals expected to be the healthiest and most fertile [3].

It follows that, in order to conclude that a face or body cue is a valid cue to health, it must be related both to perceptions of health and attractiveness, and to some aspect of real physiological health [4]. To date, valid cues to aspects of physiological health have primarily been identified in studies examining facial features. These include skin colour, which influences perceptions of health and attractiveness and also reflects a healthy diet [5], and facial adiposity, which influences perceived health and reflects objective measures of cardiac health and susceptibility to infectious disease [3].

A number of studies have examined the role of body size and shape in determining the apparent health and attractiveness of bodies [6–8]. These studies have found that a low waist to hip ratio (WHR) of approximately 0.7 [9] and a low Body Mass Index (BMI; weight scaled for height) of approximately 18–19 kg/m^2^ [10] are perceived as most attractive in female bodies, while a low waist to chest ratio (WCR) of approximately 0.7, and relatively high BMI (approximately 26) are perceived as most attractive in male bodies [11] in Western societies. More recently, it has been shown that female, but not male observers choose lower apparent BMI to optimise the attractive appearance of women’s faces than to optimise the healthy appearance of women’s faces [12]. However, both male and female observers have been found to prefer a lower apparent BMI when making attractiveness than health judgements for women’s bodies [13,14]. This effect has been attributed to exposure to, and internalization of, thin “idealised” bodies in the mass media [12], which has been shown to increase body dissatisfaction [15–17].

By contrast, the idealized male body is characterized by lean muscularity [18], and men have been found to desire, in general, a more muscular physique than that currently possessed [11,19]. However, studies have not previously addressed whether observers will choose higher male body muscle to optimize attractive appearance than to optimise healthy appearance of men’s bodies. Such a finding would suggest that a similar process of internalization of body ideals influences perceptions of male body attractiveness, as is seen in female bodies.

Previous research has asserted the importance of both body shape (e.g. WHR) and size (e.g. BMI) [10,13,14], but has not addressed the role of fat and muscle composition in determining these size and shape preferences. The reliance on BMI as a measure of body size in research addressing the attractiveness and health of bodies is problematic for two main reasons. First, BMI does not differentiate between fat and muscle, such that two individuals with the same BMI may have very different levels of fat and muscle in their bodies. A recent study found that 29% of people classified as lean using a traditional BMI scale had levels of body fat usually associated with obesity [20]. Conversely, individuals with high muscle mass may be incorrectly classified as obese when using BMI as an index of weight status [21]. Second, the formula underestimates the risk of obesity-related morbidity in shorter people and overestimates this risk in taller people [22].

Previous research has shown that high body fat is associated with a range of negative health outcomes, including diabetes [23], cardiovascular disease [24], myocardial infarction [25] and restricted movement [26]. Very low fat mass can also be deleterious to health, however, particularly in women. On average, women have a greater percentage body fat—needed for ovulation and subsequent fertility, gestation and lactation—than men [27,28]. Women with very low or very high amounts of fat mass are less likely to ovulate and be fertile [29–32], possibly due to the resulting hormonal imbalance [33]. Similarly, men with high levels of body fat experience reduced fertility [34,35]. It may be predicted, therefore that, if attractiveness is a mechanism for identifying healthy, fertile mates, healthy levels of body fat (21–33% for women and 8–21% for men; [36]) will be perceived as healthiest and most attractive.

Men have approximately 60% more muscle mass than women [27,28]. High muscle mass in men is associated with various positive health outcomes, including increased physical fitness, longevity [2,37] and a decreased risk of developing some diseases [38]. High muscle mass in men is also associated with indicators of mating success, including positive body image [19,39,40], and a higher number of sexual partners [28]. Conversely, very high levels of muscularity are associated with up to 50% higher dietary energy requirements [28], and extreme testosterone levels, which increase with muscle mass, and are associated with poor immune system activity [28,41].

Here, we examine the impact of fat and muscle mass on the apparent health and attractiveness of men’s and women’s bodies, by allowing male and female observers to manipulate the shape of bodies along empirically-derived fat and muscle dimensions to optimise their healthy and (separately) attractive appearance.

It is hypothesised that (i) if the evolutionary conception of attractiveness as a mechanism for identifying healthy partners explains observers’ perceptions of healthy and attractive body fat and muscle, these perceptions will be in line with physiologically healthy values in male and female bodies. (ii) If internalization of attractive body ideals impacts on perceptions of attractiveness more than on perceptions of health, observers will choose lower fat mass to optimise attractive appearance than to optimise healthy appearance in women’s bodies, and will choose higher muscle mass to optimise attractive appearance than to optimise healthy appearance in men’s bodies.

## Method

All work was approved by the Macquarie University Human Research Ethics Committee. All participants gave prior, informed consent in writing.

### Participants

For the stimulus acquisition phase, participants were 192 individuals (128 females), of mean age 20.76 years (SD = 5.35) recruited via the university undergraduate participant pool, and advertisements around campus. Recruitment was restricted to Caucasian participants between ages 18–30 to minimise the potentially confounding effects of age and ethnicity [42]. Stimulus acquisition participants will henceforth be referred to as “subjects” to distinguish them from experimental participants, who will be referred to as “observers”. Subjects received course credit or AU$20 for their time.

Observers were 63 individuals (33 males) comprising first-year undergraduate psychology students and respondents to study advertisements (mean age = 21.12, SD = 3.21). Observers received course credit or AU$10 for their time.

### Procedure

#### Stimulus acquisition

**Fig 1:**
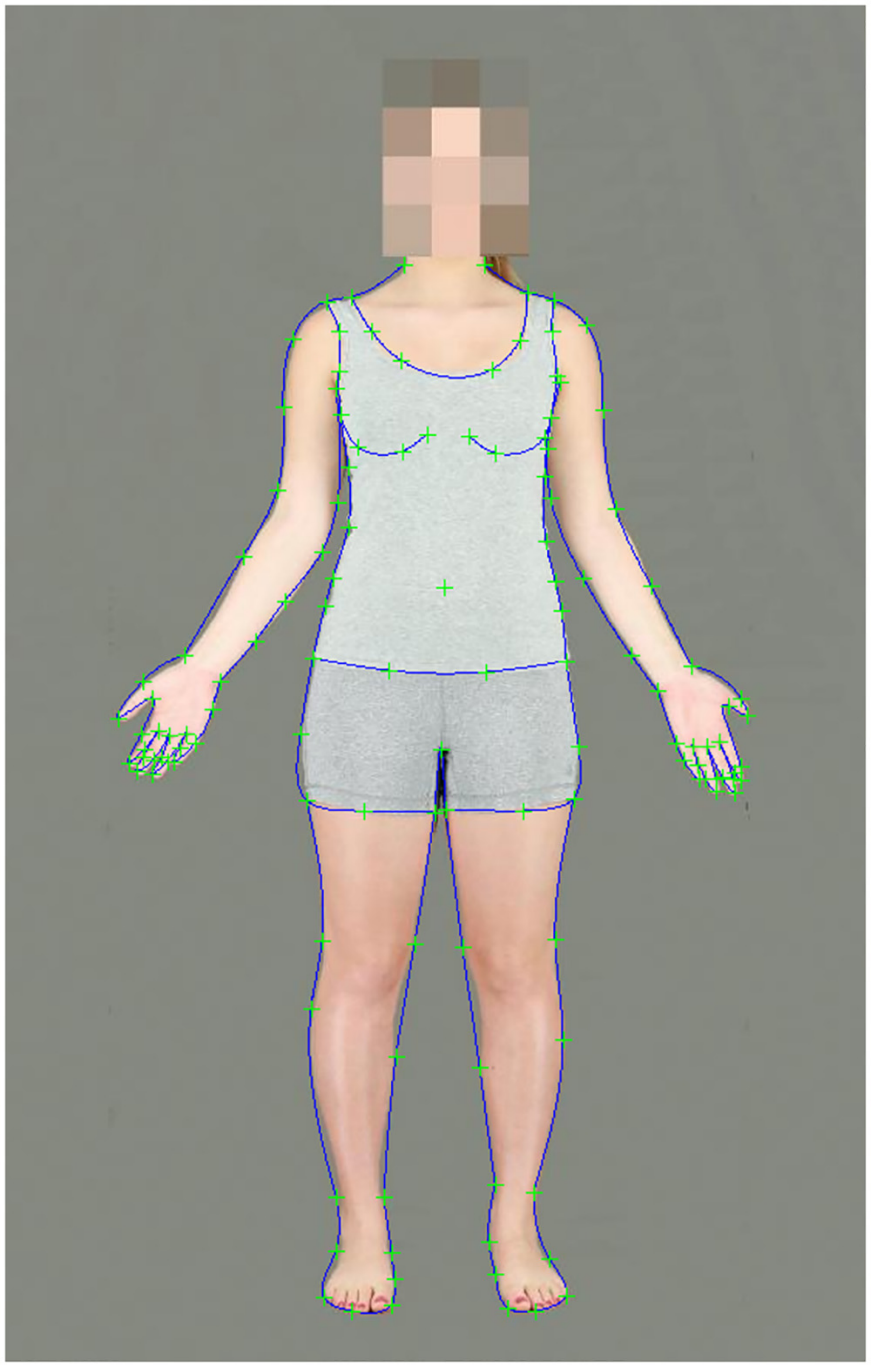
A subject standing in the frontal anatomical position. The body image has been delineated using the 130 landmark points. Face obscured to meet ethical requirements of confidentiality.

Subjects completed a simple demographic questionnaire, in which they were asked to provide their age, sex and ethnicity, before their height was measured using a standard tape measure. A Tanita SC-330 Body Composition Analyser (Tanita, Tokyo) was used to measure muscle and fat mass [43]. Subjects were then photographed using an EOS 50D camera (Canon, Oita) with an 18—55mm lens, fitted onto a tripod one metre above the ground. The subject stood within a 117 x 90 x 210cm booth situated 3 metres away from the camera, painted with Munsell N5 standard grey paint, and illuminated using 15 d65 daylight simulation fluorescent tubes in high frequency fixtures to reduce the effects of flicker. Light was diffused using Perspex diffusers and no other light source was present in the room. Subjects wore a grey singlet and shorts, which were tight fitting to ensure the visibility of body shape while maintaining appropriate modesty. Subjects were photographed in the frontal anatomical position to control for possible confounding effects of posture, which may interact with body size to affect attractiveness [44,45], and were instructed to stand with their heels in front of a line marked in the middle of the photography booth (Fig 1).

#### Stimulus Manipulation

Photographs were resized to 600 x 900 pixels and bodies were delineated with 130 landmark points and aligned to ensure they are upright and in the middle of the screen, using PsychoMorph [46] (Fig 1).

The primary purpose of the stimulus manipulation phase was to create separate visual continua of fat content and muscularity, which could be altered by observers. Since larger individuals tend to have higher absolute fat and muscle mass and smaller individuals tend to have lower absolute fat and muscle mass, linear regression was used to control for these confounding factors. A linear regression was run, with fat mass as the dependent variable and muscle mass and height as the independent variables. Unstandardised residuals were saved and used as a measure of fat mass controlling for muscle mass and height. A second linear regression was run with muscle mass as the dependent variable and fat mass and height as independent variables. Unstandardised residuals were saved and used as a measure of muscle mass controlling for fat mass and height.

PsychoMorph was used to create a composite of the 10 female subjects with the highest fat residual score (“female high fat”), and composites of the 10 with the lowest fat residual score (“female low fat”), the 10 with the highest muscle residual scores (“female high muscle”) and the 10 with the lowest muscle residual scores (“female low muscle”) were produced, ensuring that individual subjects did not appear in both high and low fat or muscle composites. This process was repeated for the creation of the male endpoint composite images. Due to the smaller number of male subjects recruited for the stimulus acquisition phase, only the 5 subject images at each extreme were used to create these averages. Significant differences were seen between the subjects in the high and low endpoint composites in the dimension being manipulated, but not in the dimensions being controlled (Table 1), indicating that fat and muscle dimensions had been successfully separated for both male and female composites.

**Table 1:** Differences in fat and muscle mass and body height of subjects in the endpoint average images, and results of independent samples t-tests.

Sex	Dimension manipulated	Dimension	Low mean	High mean	Mean difference
**Female**	**Fat**	Fat	10.56kg	22.60kg	12.04kg***
		Muscle	43.39kg	41.89kg	-1.50kg (n.s.)
		Height	166.56cm	164.29cm	-2.27cm (n.s.)
	**Muscle**	Fat	16.69kg	13.95kg	2.74kg (n.s.)
		Muscle	39.38kg	44.59kg	5.21kg***
		Height	163.74cm	165.28cm	1.54cm (n.s.)
**Male**	**Fat**	Fat	7.06kg	24.40kg	17.34kg*
		Muscle	58.28kg	56.48kg	-1.80kg (n.s.)
		Height	177.16cm	175.20cm	-1.96cm (n.s.)
	**Muscle**	Fat	15.20kg	17.40kg	2.20kg (n.s.)
		Muscle	49.54kg	67.48kg	17.94kg*
		Height	175.40cm	176.90cm	1.50cm (n.s.)

* p < .05,

*** p <.001

An image of a single female subject was then selected from the remaining pool of images. The high and low fat average female images produced above were used as endpoints, and the subject image was transformed by 115% (chosen to maximise the magnitude of the transform without introducing artefacts to the images) of the difference between the two end-points, using PsychoMorph in a sequence of 13 frames (labelled 0 to 12). Thus, identity cues were maintained, and frame 0 was reduced in apparent fat mass by 13.85kg, increasing in equidistant steps such that frame 6 was the original subject image, and frame 12 was increased in apparent fat mass by 13.85kg.

**Fig 2:**
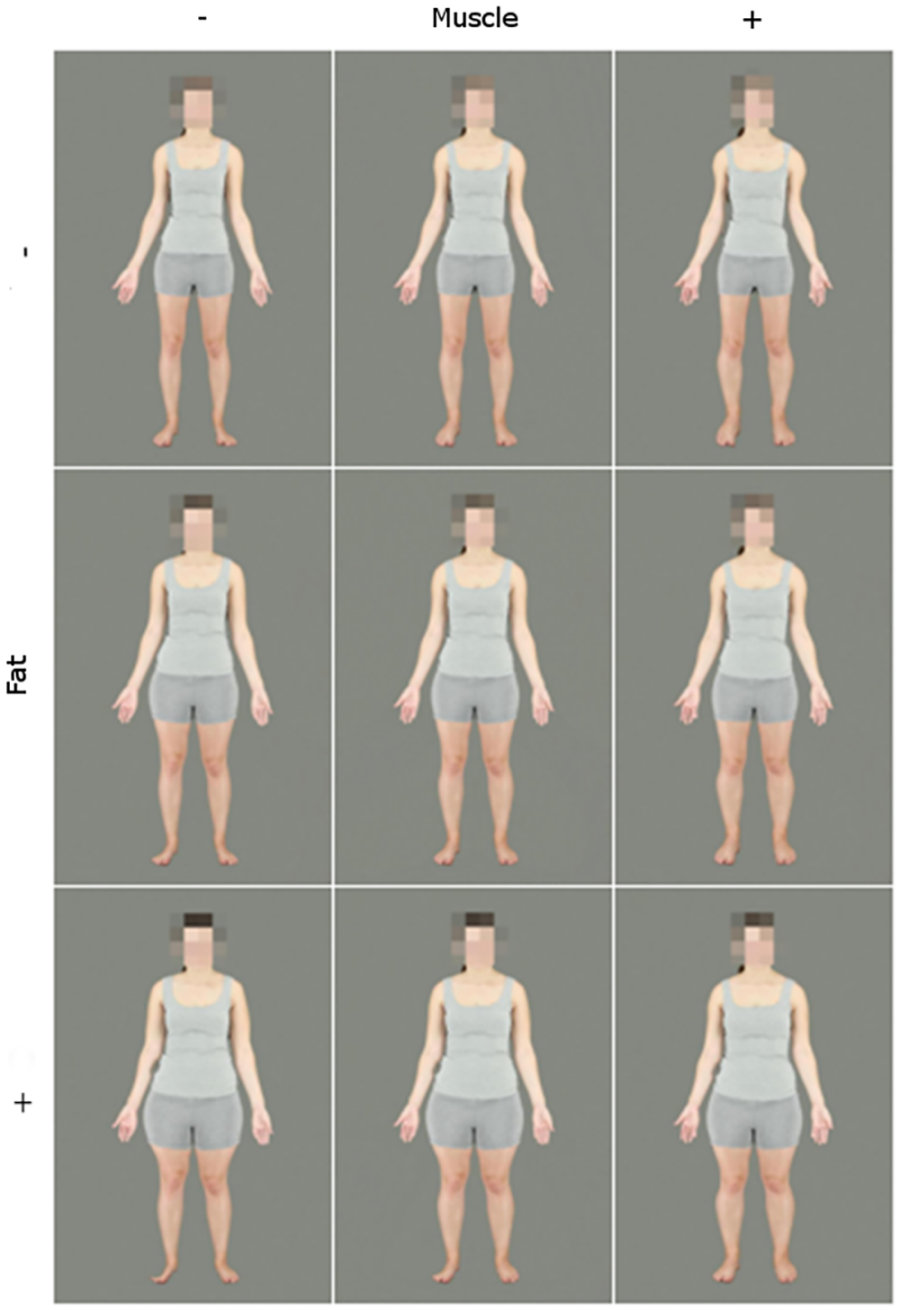
A female identity transformed across levels of fat and muscle continua. This figure depicts the end- and mid-points of the transformations. Left to right, top to bottom: low fat, low muscle; low fat, original muscle; low fat, high muscle; original fat, low muscle; original fat, original muscle; original fat, high muscle; high fat, low muscle; high fat, original muscle; high fat, high muscle.

**Fig 3:**
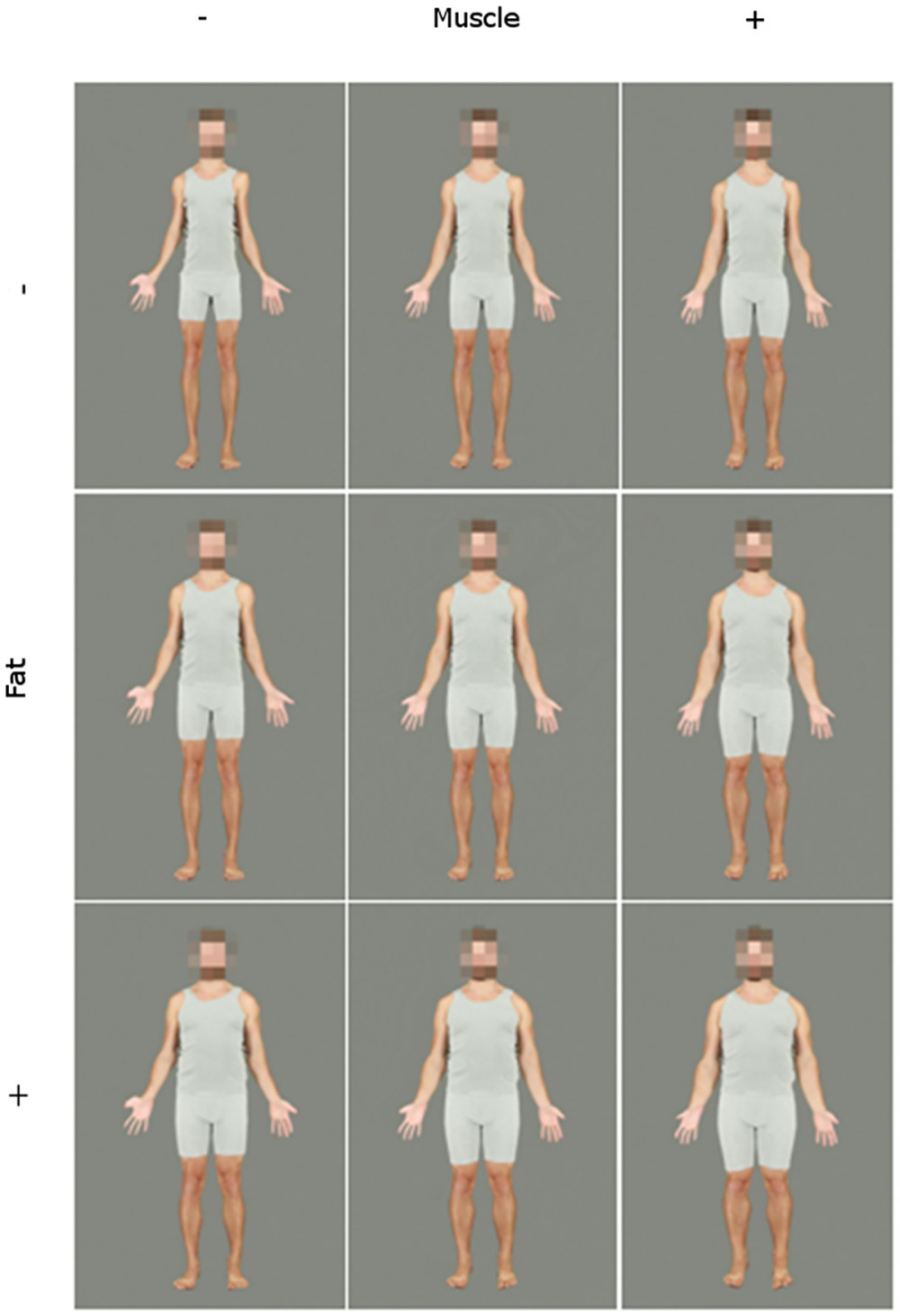
A male identity transformed across levels of fat and muscle continua. This figure depicts the end- and mid-points of the transformations. Left to right, top to bottom: low fat, low muscle; low fat, original muscle; low fat, high muscle; original fat, low muscle; original fat, original muscle; original fat, high muscle; high fat, low muscle; high fat, original muscle; high fat, high muscle.

Each of the 13 fat transformed frames was then manipulated in a sequence of 13 frames (labelled 0 to 12) by 115% of the difference between the high and low muscle end-points, such that frame 0 was reduced by 5.99kg of apparent muscle mass, increasing in equidistant steps so that frame 6 was not adjusted in apparent muscle mass and frame 12 was increased by 5.99kg of apparent muscle mass. Thus, 169 frames were produced for the subject identity that varied from -13.85kg to +13.85kg from the original fat mass in one dimension, and from -5.99kg to +5.99kg from the original muscle mass in the other dimension (Fig 2). This process was repeated for a further 14 randomly selected female and 15 randomly selected male subject identities. Only subject identities that fell within 1SD of the mean in fat and muscle mass were used, and both male (*M* = 17.29%, SD = 4.07) and female (*M* = 22.33%, SD = 2.57) subject identity groups fell within the normal range of body fat in the population. Male identities varied from -17.34kg to +17.34kg from the original fat mass and from -17.94kg to +17.94kg of the original muscle mass (Fig 3).

Faces were blurred using the Photoshop pixelate mosaic function (which averaged pixel colour over a square 45x45 pixels in size), to render individuals’ faces unidentifiable and to obscure facial shape changes introduced by the transforms.

#### Data collection

Observers were presented with the subject identities one at a time in an applet developed in Matlab version 2014a, operating the Psychophysics Toolbox. The applet allowed observers to manipulate the apparent muscle and fat mass of the bodies presented to them. Moving the mouse along the horizontal axis (left-right) adjusted apparent fat mass while moving vertically (up-down) adjusted apparent muscle mass by cycling through the frames of the transform. The starting position of the cursor on the screen was randomised before each trial and the mouse cursor was hidden so observers could not easily identify a midpoint. Prior to the start of the experiment, observers were verbally told that they would see some bodies, and that moving the mouse horizontally would change the bodies in one way, while vertical movement changed the bodies in another way. They were informed that there would be two types of trial, one where they should make the bodies look as healthy as possible, and one where they should make the bodies as attractive as possible. Clicking the left mouse button saved the data and presented the next trial. Observers were not told the nature of the transforms, and “healthy” and “attractive” were not defined. Trials were blocked by sex of the subject and by trial type. Before each block, on-screen instructions asked the observers to “Make the body as HEALTHY as possible” (health trials) or “Make the body as ATTRACTIVE as possible” (attractiveness trials).

### Statistical methods

For each trial, the coordinates of the frame chosen were saved, such that each trial had a score from 0–12 in the fat dimension and a score from 0–12 in the muscle dimension. Six was subtracted from each score, and they were then divided by 6, converting the scores to a scale from -1 to +1, where a negative fat score represented a reduction in apparent fat mass, a positive score represented an increase in apparent fat mass, and 0 represented no change. Equivalent transformations were made to muscle scores. These transformed scores were multiplied by the values in Table 1 to give an amount of fat and muscle change in kg. Change scores were then added to the measured fat and muscle mass of the subject identity in the trial to give an optimal fat and muscle mass chosen to optimise health/attractiveness in each trial. Scores were averaged across subject identities for each observer separately for health and attractiveness trials.

The fat/muscle mass (kg) chosen by each observer was used as the dependent variable. A 4-way mixed ANOVA was performed. Within-subject independent variables were composition dimension (fat or muscle), sex of body (male or female) and trial type (healthy or attractive), while the between-subjects factor was sex of the observer.

## Results

A significant main effect of composition was found, F(1,61) = 5158.91, p < .001, $\eta_{p}^{2}$ = .99, with observers choosing significantly higher muscle mass (*M* = 53.19kg, SD = 2.55) than fat mass (*M* = 11.26, SD = 3.85), in line with the composition of the human body.

A significant main effect of rating was found, with observers choosing a lower combined fat and muscle mass for attractiveness (*M* = 64.03kg, SD = 4.64) than for health (*M* = 64.87kg, SD = 4.98) trials, F(1,61) = 6.27, p = .015, $\eta_{p}^{2}$ = .09.

A significant main effect of body sex was found, F(1,61) = 1629.26, p < .001, $\eta_{p}^{2}\;=\;.96,$ with observers choosing a lower combined fat and muscle mass for female (*M* = 52.76kg, SD = 4.82) than for male bodies (*M* = 75.42kg, SD = 5.40), in line with the differing body weights of the sexes.

The interaction between trial type and observer sex was found to be significant, F(1,61) = 4.26, p = .043, $\eta_{p}^{2}$ = .07. Paired samples t-tests showed that, while female observers chose a lower combined fat and muscle mass for attractiveness (*M* = 65.07kg, SD = 4.01) than for health trials (*M* = 66.73kg, SD = 3.84; t(29) = 3.13, p = .004), male observers did not show a significant difference between attractiveness (*M* = 63.08kg, SD = 5.01) and health (*M* = 63.17kg, SD = 5.34) trials, t(32) = .17, p = .86. Independent samples t-tests showed that, while men (*M* = 63.08kg, SD = 5.01) and women (*M* = 65.07kg, SD = 4.01) did not differ significantly in the amount of combined fat and muscle mass chosen in attractiveness trials, t(61) = 1.72, p = .090, women (*M* = 66.73kg, SD = 3.84) chose significantly higher combined fat and muscle mass than men (*M* = 63.17kg, SD = 5.34) in health trials, t(61) = 3.01, p = .004.

We found a significant interaction between observer sex and body sex, F(1,61) = 5.58, p = .021, $\eta_{p}^{2}$ = .08. Both male, t(32) = 31.08, p < .001, and female, t(29) = 56.55, p < .001, observers chose a lower combined fat and muscle mass for female bodies than for male bodies, in line with the difference in body weight between the sexes. However, while male observers (*M* = 73.49kg, SD = 5.37) chose a lower combined body mass for male bodies than did female observers (*M* = 77.55kg, SD = 4.64; t(61) = 3.20, p = .002), no significant difference was found between male (*M* = 52.09kg, SD = 5.33) and female observers (*M* = 53.49kg, SD = 4.15) in their preferred combined fat and muscle mass for female bodies, t(61) = 1.16, p = .25.

A significant interaction was found between composition dimension and body sex, F(1,61) = 645.49, p < .001, $\eta_{p}^{2}$ .91. Observers chose lower fat mass than muscle mass for both male, t(62) = 58.67, p < .001, and female, t(62) = 75.09, p < .001, bodies, in line with the usual composition of human bodies. Observers chose slightly lower fat mass for female (*M* = 10.31kg, SD = 3.84) than male (*M* = 12.16kg, SD = 4.69) bodies, t(62) = 3.81, p < .001) and much lower muscle mass for female (*M* = 42.45kg, SD = 1.63) than male (*M* = 63.27kg, SD = 4.06) bodies, t(62) = 45.60, p < .001, reflecting the fact that, for their size, observers chose a higher proportion of fat mass for female than male bodies.

A significant 3-way interaction was found between composition dimension, trial type and body sex, F(1,61) = 5.63, p = .021, $\eta_{p}^{2}$ = .08. In order to further explore this 3-way interaction, the data was split by body sex, and separate 2-way ANOVAs were run on the male and female data separately.

**Fig 4:**
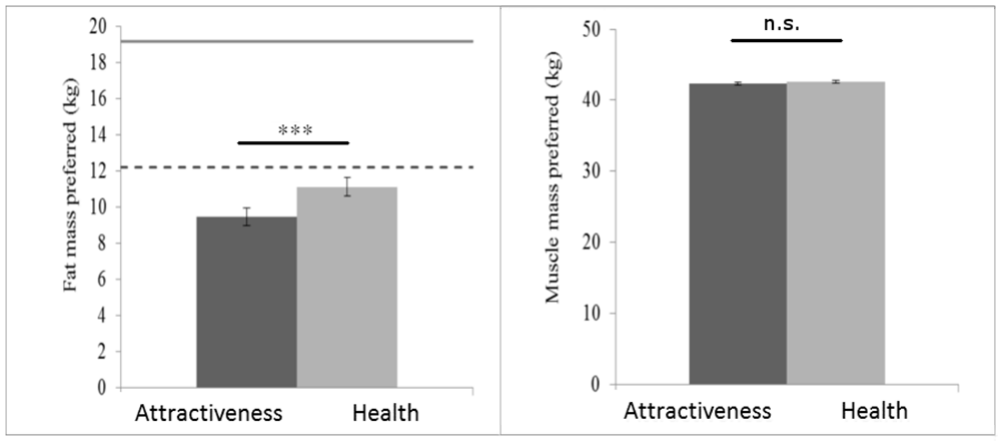
Preferred body fat and muscle mass of women’s bodies. Observers prefer lower fat mass to optimise the attractive appearance of women’s bodies than to optimise the healthy appearance of women’s bodies (left). No difference is found in the amount of muscle mass chosen to optimise the attractive and healthy appearance of women’s bodies (right). Error bars show standard error of the mean. Dashed line shows lower bound of healthy fat mass (kg) and solid line shows the upper bound of healthy fat mass (kg) for young women with the body mass of the average female subject identity in this study. *** p < .001.

For the female bodies, a significant main effect of composition dimension was found, F(1,62) = 5638.30, p < .001 $\eta_{p}^{2}$ = .99, reflecting the fact that observers chose lower body fat (*M* = 10.31kg, SD = 3.84) than body muscle (*M* = 42.45kg, 1.63), in line with the usual composition of the human body. A significant main effect of trial type was also found, F(1,62) = 20.26, p < .001, with observers choosing a lower combined fat and muscle mass for attractiveness (*M* = 51.79kg, SD = 5.26) than for health (*M* = 53.72kg, SD = 4.95) trials. A significant interaction was found between composition dimension and trial type, F(1,62) = 13.48, p = .001, $\eta_{p}^{2}$ = .18. Observers chose lower fat mass for attractiveness (*M* = 9.48kg, SD = 4.02) than for health (*M* = 11.14kg, SD = 4.13) trials, t(62) = 4.80, p < .001, but no significant difference was found in amount of muscle mass chosen for attractiveness (*M* = 42.32, SD = 1.90) and health (*M* = 42.58kg, SD = 1.76) trials, t(62) = 1.26, p = .211 (Fig 4).

**Fig 5:**
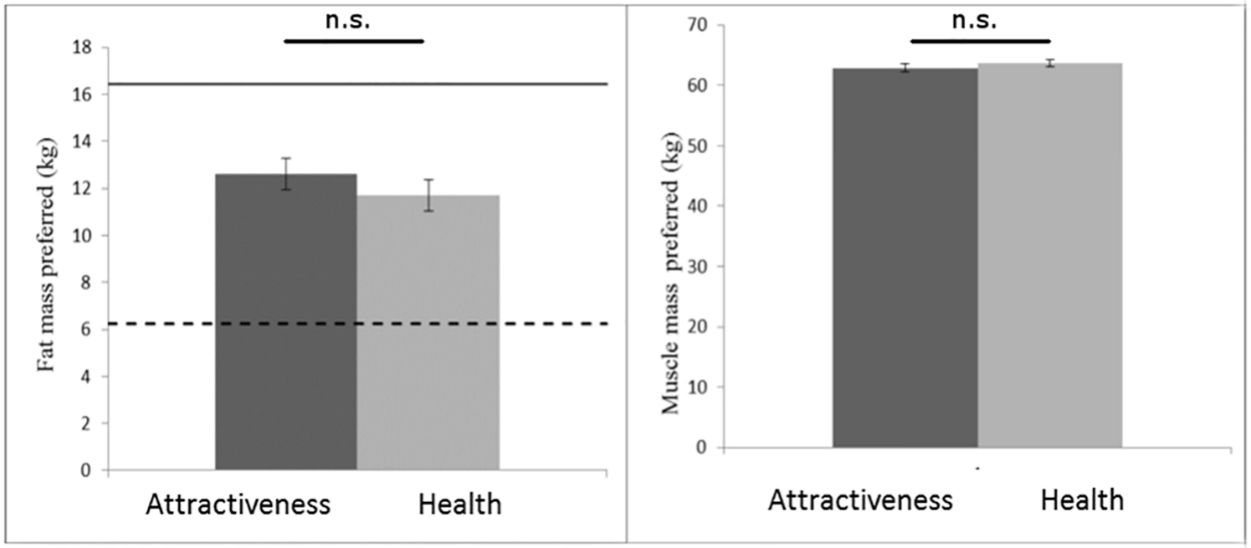
Preferred body fat and muscle of men’s bodies. Observers did not differ in the amount of fat mass (left) or muscle mass (right) they preferred to optimise the healthy and attractive appearance of male bodies. Error bars show standard error of the mean. Dashed line shows lower bound of healthy fat mass (kg) and solid line shows the upper bound of healthy fat mass (kg) for young men with the body mass of the average male subject identity in this study.

For the male bodies, a significant main effect of composition dimension was found, F(1,62) = 3449.27, p < .001 $\eta_{p}^{2}$ = .98, reflecting the fact that observers chose lower body fat (*M* = 12.16kg, SD = 4.69) than body muscle (*M* = 63.27kg, 4.06), in line with the usual composition of the human body. No main effect of trial type, F(1,62) = 0.7, $\eta_{p}^{2}$ = .00, or interaction between trial type and composition dimension, F(1,62) = 2.12, p = .150, $\eta_{p}^{2}$ = .03 were found (Fig 5).

No other significant main effects or interactions were found: composition * observer sex, F(1,61) = .73, p = .396, $\eta_{p}^{2}$ = .01; composition dimension * trial type, F(1,61) = .02, p = .886, $\eta_{p}^{2}$ = .00; composition dimension * body sex * observer sex, F(1,61) = .11, p = .737, $\eta_{p}^{2}$ = .00; trial type * body sex * observer sex, F(1,61) = .12, p = .735, $\eta_{p}^{2}$ = .00; composition dimension * trial type * body sex * observer sex, F(1,61) = .36, p = .552, $\eta_{p}^{2}$ = ..01.

**Fig 6:**
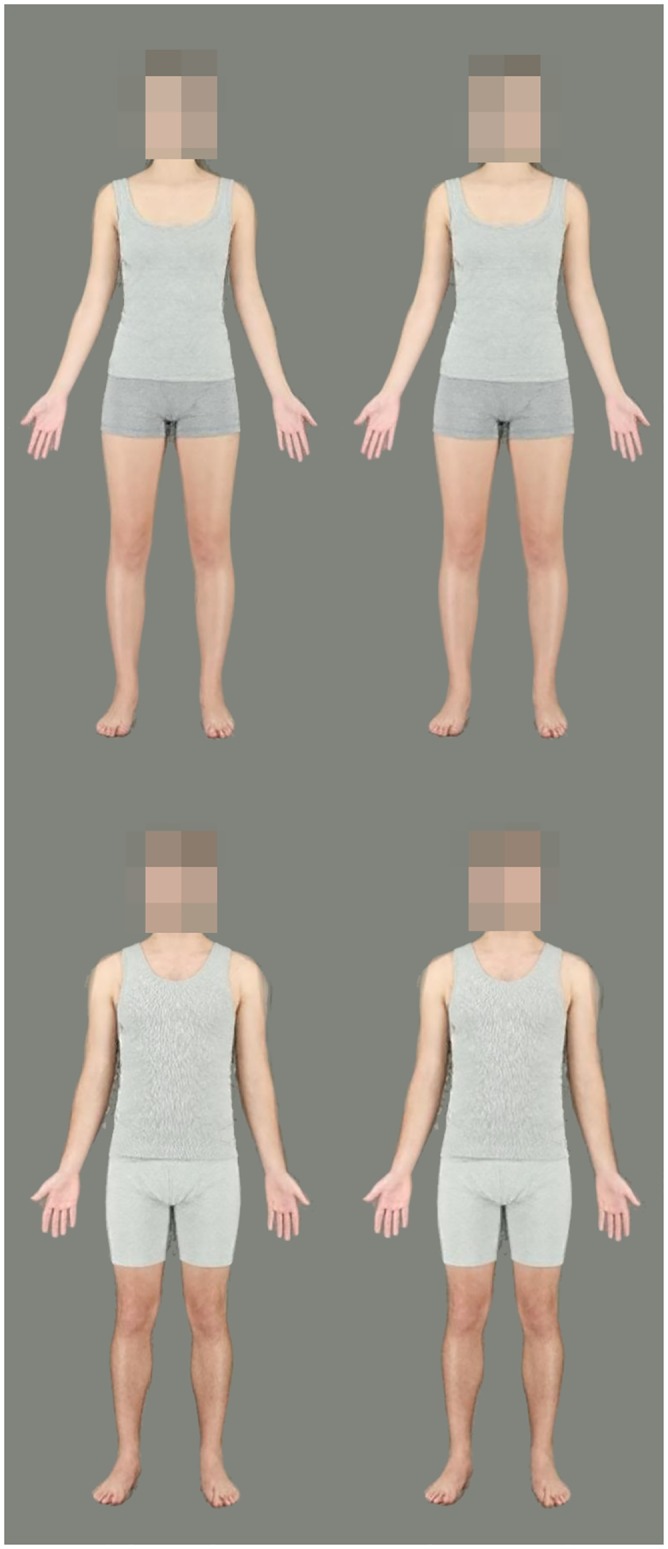
Average preferred body composition of men’s and women’s bodies. Left images show fat and muscle mass chosen to optimise attractiveness. Right images show fat and muscle mass chosen to optimise health. Top row female bodies, bottom row male bodies.

Given that the average weight of female subjects presented to observers was 58.14kg, one sample t-tests showed that the average fat mass chosen to optimise the attractive (16.31%, t(62) = 2.06, p = .044) and healthy appearance (19.16%, t(62) = 5.40, p < .001) were significantly below the healthy range of 21–33% for a young Caucasian woman [36]. However, given that the mean weight of male subjects presented to observers was 79.29kg, the mean fat mass chosen for male bodies fell within the healthy range of 8–21% [36,38] for both attractiveness (15.89%) and health (14.75%) trials. Average body composition chosen to optimise attractive and healthy appearance of men’s and women’s bodies are shown in Fig 6.

## Discussion

Observers chose a lower fat mass to optimise the attractive appearance than they chose to optimise the healthy appearance of women’s bodies. This difference was not seen in the muscle mass chosen to optimise the healthy and attractive appearance of women’s bodies. This suggests that the previously observed phenomenon of preferring lower female body size for attractive than healthy appearance [12,13] is driven by preference for lower fat mass, rather than by lower muscle mass or by lower body size *per se*. While it was predicted that observers would choose a low apparent fat content of female bodies to optimise attractive appearance, the amount of fat mass chosen was lower than expected. The amount of fat chosen to optimise the healthy and attractive appearance of female bodies in this study fell significantly below the established healthy range (21–33% for young Caucasian women [36]). This is in agreement with previous studies on healthy and attractive BMI, which also placed attractive and healthy-looking female body mass below [13,14] or at the very bottom of [11] the physiologically healthy range. No difference was found between male and female observers’ preferences for muscle and fat mass in female bodies, in line with previous findings using BMI [11,14].

Observers’ preference for low fat mass in women may reflect internalization of the “thin ideal” associated with repeated exposure to images of thin women in the popular media [12,15–17]. While measures of internalization of the thin ideal, and participants’ own BMI [14] were not taken in the current study, previous studies have suggested that these individual differences variables may impact on participants’ perceptions of healthy and attractive body composition [12,14–17]. This question should be addressed in future studies.

It should be noted that observers were undergraduate students recruited from a highly urbanised and affluent region of Australia. It is well known that the preference for thinness as an “ideal” body type is more pronounced in middle-high socio-economic groups [7,15], and in more developed and urbanised environments [15,42]. This observation has been taken to infer the acquisition of norms of attractiveness that reflect local preferences in terms of perceived weight-related fertility and health advantages [42,47]. It would be interesting to see whether this result could be replicated in a more diverse sample.

Alternatively, the preference for lower body weight to optimise women’s attractiveness than healthy appearance may reflect a preference for youthful women [13], since youth is associated with higher reproductive value (expected future reproductive output) in women [48]. Body fat increases across the lifespan [49], while muscle mass remains approximately stable until age 45 [50].

Male body perception research dating as early as the 1950s has suggested that greater levels of muscle mass are preferred in the male body (for a review of the literature, see [51]). In the present study, this would have been congruent with a high level of muscle mass for attractiveness and health ratings. A drive for muscularity has been found in a cross-cultural study [40] in which 90% of North American males expressed a desire for increased muscle mass, as did two thirds of Ukrainian men and approximately half of the Ghanaian men in the study sample. More recently, there has been a preference for lean muscularity (increased muscle mass and reduced fat mass), rather than muscularity *per se*, among males in developed nations [18,52]. When taken to an extreme, this preference, like that for the thin ideal in women, is associated with increased risk for eating disorder and comorbid psychopathology [18,19,40,53]. This preference for lean muscularity is seen in our results. However, for men’s bodies, internalization of this ideal does not appear to have driven a difference in the ideal body fat and muscle preferred for attractive, compared to healthy appearance. While the use of a relatively small, non-clinical sample in the current study precludes drawing any implications of the findings for clinical practice, it would be of interest to replicate these findings in a much larger sample of young men, in which a subgroup of participants with clinically significant body image disturbance could be identified, and/or a clinical sample of males with such disturbance (cf. [18,52]). Research of this kind may be beneficial in informing treatment approaches, the extent to which attention should be given to preoccupation with leanness as opposed to preoccupation with muscularity in treating males with eating disorders and/or muscle dysmorphia for example [52].

Also of note is that observers adjusted both the attractive and healthy male body figures to consist of approximately 16% fat and 80% muscle, given a mean body weight of 78.24kg for the subject identities. The figure of 16% lies within the healthy fat range of 8–21% for young adult Caucasian males [36,38]. While the World Health Organisation does not release recommendations pertaining to healthy or ideal muscle mass percentages, observers’ preference for approximately 80% muscle appears reasonable given that this represented an increase of only 0.55kg above the original muscle mass of the subjects in the study. Our results suggest that, in men’s bodies, what is perceived as attractive and healthy is closely aligned with what is physiologically healthy. These results are consistent with the evolutionary view that attraction is a mechanism for identifying a healthy mate [1–3].

Male observers in the current study preferred a lower male body mass for both health and attractiveness trials than did female observers. Although one early and one more recent study found no differences in preferences for male body size and shape as a function of observer sex [11,54], evidence in this regard is limited and these discrepant findings warrant further investigation.

While observers chose muscle mass in the normal range to optimise the healthy and attractive appearance of men’s bodies, there is evidence that men with lower WCR are perceived as more physically dominant and better protectors [55]. This suggests that muscle mass may primarily be perceived as a cue to physical formidability. Indeed, a recent study has suggested that the relatively much higher muscle mass in men than in women (men have approximately 60% greater muscle mass than women, despite being only 15–20% larger in terms of overall body mass) is better explained as an evolved adaptation for competition with other men than as an ornament for attracting women [27].

There is evidence that lower weight individuals are more likely to overestimate, while higher weight individuals are more likely to underestimate their own body weight [56]. However, observers’ own BMI appears to have little to no effect on their perceptions of what constitutes a healthy or attractive weight assessed from other people’s faces [12] or bodies [14]. It would be interesting to see future studies address how observers’ own body fat and muscle mass relates to their perception of healthy and attractive body composition. It is also noteworthy that, as predicted by Weber’s law, just noticeable differences become larger as the BMI of the target body gets higher [57]. In the current study, however, the body composition that was selected to optimise the healthy and attractive appearance of male and female bodies did not extend into the very large part of the distribution, with participants choosing apparent fat content in line with the healthy range for men, and below the healthy range for women’s bodies. While observers did choose to increase the apparent muscle mass of men’s and women’s bodies, these increases were relatively small (less than 1kg increase over the initial size of the bodies). This suggests that lack of perceptual sensitivity introduced by very large stimuli is unlikely to have introduced significant error into our results.

Previous studies have shown that images of bodies apparently in motion (walking) are perceived as more attractive than bodies at rest (standing still) [44,45], possibly because of an accentuation of WHR in women [44], or because they appear more emotionally positive [45]. In the current study, we controlled for possible confounding effects of posture and motion by capturing all stimuli in a standard anatomical position. However, future studies should investigate whether perceived motion interacts differently with apparent muscle mass and apparent fat mass.

### Conclusion

We examined the role of body fat and muscle mass on the apparent health and attractiveness of male and female bodies. The amount of fat mass chosen to optimise the healthy and attractive appearance of women’s bodies was slightly below the healthy range. Both male and female observers chose significantly less fat mass to optimise the attractiveness of women’s bodies than to optimise the healthy appearance of women’s bodies. This suggests that previous studies’ findings of lower BMI preference to optimise attractiveness than to optimise healthy appearance of women’s bodies is driven by a preference for lower fat, rather than lower muscle mass. It is also consistent with the hypothesis that internalization of the thin ideal in affluent Western populations may be driving the attractiveness preferences for women’s bodies, and future studies should examine the role of internalization of thin ideals in determining perceptions of ideal healthy and attractive body composition.

For men’s bodies, the amount of muscle and fat mass chosen to optimise attractiveness was similar to the amount chosen to optimise healthy appearance, and was in line with a physiologically healthy body composition. This is in line with the lean muscularity ideal for men’s bodies, and is also in line with the hypothesis that attraction is a mechanism for identifying a healthy mate [1].

A major contribution of the present study is its validation of the use of body composition in body perception research, and the separate manipulation of apparent body fat and muscle in photographic images. Differences between the most attractive female body and the healthiest looking female body were dependent on fat, rather than muscle mass. While past research has revealed lower BMI preferences for optimising attractiveness than for optimising healthy appearance, the present study suggests that fat content accounts for a large proportion of this variance. This study also used a technique of stimulus manipulation that calculates and applies the change vectors separately for each of the 130 landmark points on the body. This is an improvement over methodologies that use simple width expansion and contraction as a proxy for changes in body size (e.g. [58,59]), since it has been shown that the rate of linear shape change associated with increased body size (BMI) varies across locations on the body [11,60] (though it should be noted that these rates of change remain approximately constant across a wide range of body size [11,60]). Thus, our stimuli simulated empirically-derived shape and size changes associated with changes in fat and muscle composition. This allows us to examine the impact of physiologically-relevant shape changes, rather than examining the impact of more geometrically-defined shape variation, such as waist-to-hip ratio or waist to chest ratio.
